# Interplay between Jahn–Teller Distortions and Structural Phase Transitions in Ruddlesden–Poppers

**DOI:** 10.1021/jacs.5c00459

**Published:** 2025-02-18

**Authors:** Anna Herlihy, Wei-Tin Chen, Clemens Ritter, Yu-Chun Chuang, Mark S. Senn

**Affiliations:** † Diamond Light Source, Harwell Campus, Oxfordshire, OX11 0DE, U.K.; ‡ Center for Condensed Matter Sciences and Center of Atomic Initiative for New Materials, National Taiwan University, Taipei 10617, Taiwan; § Taiwan Consortium of Emergent Crystalline Materials, Ministry of Science and Technology, Taipei 10622, Taiwan; ∥ Institut Laue-Langevin, 71 Avenue des Martyrs, CS20156, 38042 Grenoble Cédex 9, France; ⊥ National Synchrotron Radiation Research Center, Hsinchu, 300092, Taiwan; # Department of Chemistry, National Taiwan University, Taipei 10617, Taiwan; 7 Department of Chemistry, University of Warwick, Gibbet Hill, Coventry, CV4 7AL, U.K.

## Abstract

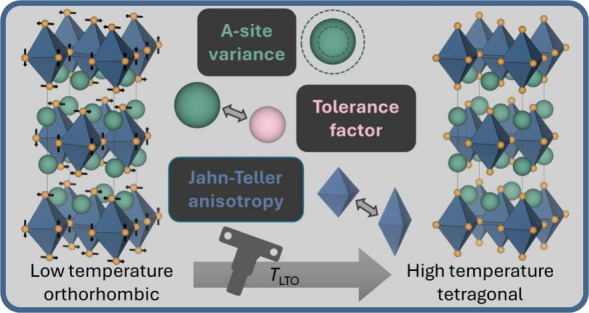

The interplay between crystallographic symmetry, structural distortions, and the tolerance factor derived from the isotropic ionic radii of the constituent cations and anions of inorganic perovskites and related materials is a ubiquitous concept in solid-state and materials chemistry. Here we demonstrate a model for the phase transition temperatures associated with these structural distortions in layered perovskites by considering the anisotropy associated with cations, which are susceptible to first-order Jahn–Teller distortions. These symmetry-lowering phase transitions are known to have a significant interplay with superconductivity in the high-*T*_C_ layered cuprates, and untangling the chemistry that can effectively control them is of the utmost relevance in the search for similar phenomena in the nickelates, the study of which has been greatly stimulated by recent reports of high-pressure superconductivity.

The Goldschmidt tolerance factor^1^ (τ = ) has been used to predict the phase stability of ABX_3_ perovskites, based on ionic radii (*r*) derived from experimental data,^2^ for almost a century. It is generally accepted that the stability range of the perovskite structure is between τ ≈ 0.71 and 1,^3^ with the lower values in this range resulting in more distorted, lower symmetry polytypes of the cubic *Pm*3̅*m* perovskites aristotype, that may be classified in terms of the kind of octahedral tilting they exhibit.^4^ The success of this metric in predicting stability windows^5,6^ and the degree and nature of the BO_6_ octahedral tilting has led to the idea being extended to so-called hybrid perovskites, in which the A-site is a molecular cation.^7,8^ In addition to considering metrics derived from the average ionic radii, it has also been shown that the second moment of the distribution (variance, *rσ*^2^) of the A-site cations can be used to explain the variation of structural and electron transition temperatures in perovskites.^9,10^

Like their perovskite counterparts, Ruddlesden–Popper (RP) A_*n*+1_B_*n*_O_3*n*+1_ oxides (Figure 1 (a)) host a remarkable set of properties including superconductivity,^11−13^ hybrid improper ferroelectricity,^14−16^ and negative thermal expansion.^17,18^ Much of the functionality is derived from or is intrinsically linked to the tilting and rotations of layers of corner-sharing BO_6_ octahedra. For example, it has been shown that the transition temperature, *T*_C_ associated with the improper ferroelectric ordering, driven by octahedral tilting and rotations in n = 2 RPs scales with tolerance factor.^19^ Therefore, controlling the nature and magnitude of these tilts and rotations is an important endeavor. Guiding principles originating from perovskite tolerance factor trends are poorly understood in RP structures in comparison to ABO_3_ perovskite counterparts, and while various authors have proposed amendments to the definition of τ,^20,21^ to reflect the varied coordination introduced by the AO rock salt layering interface, a satisfactory picture of its interplay with the rotation and tilts of the BO_6_ octahedral is still missing.^22^ One of the problems in understanding the structure–property relationship is that the RP aristotype is less constrained by symmetry than the perovskite aristotype. While this creates additional challenges in understanding structural trends, as we will show, it also provides substantial opportunities for introducing new control parameters to tune fundamental and technologically relevant structure–property relationships.

**Figure 1:**
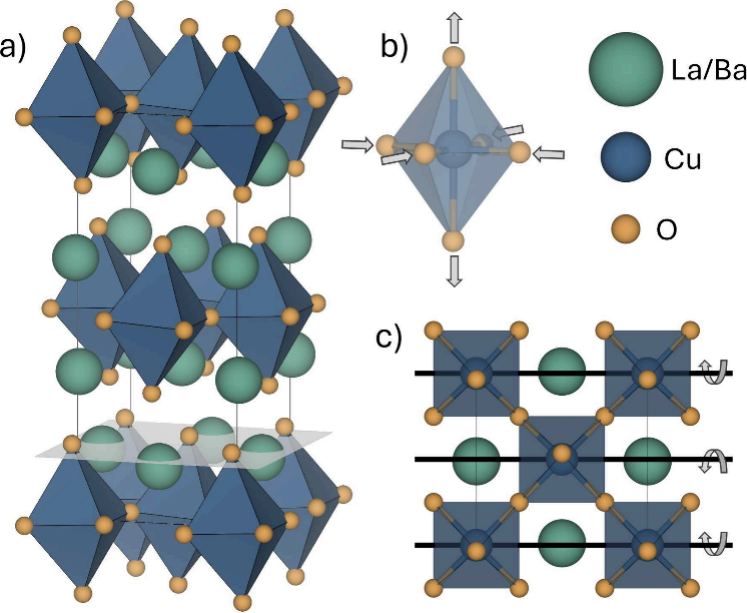
a) The crystal structure of *n* = 1 RP in the low temperature orthorhombic (LTO) phase of La_2–*x*_Ba_*x*_CuO_4_. b) The Q_3_ mode, which describes an elongated Jahn–Teller distortion. c) The sense of the octahedral tilting in the LTO phase. Experimentally observed positive rumpling with A-sites displaced toward the perovskite blocks is depicted in a).

In this communication, we demonstrate how, in possibly the most studied class of RPs, namely the n = 1 high-*T*_C_ superconducting cuprates, there is a clear interdependence between the single ion anisotropy associated with Jahn–Teller (JT) active sites and the phase transition temperatures associated with low temperature orthorhombic (LTO) octahedral tilting, that is driven by a soft-mode instability (see Figure 1). We elucidate this effect by tuning the LTO phase transition temperature (*T*_LTO_) in a series of compounds via the substitution of JT-active Cu^2+^ with JT-inactive Mg^2+^. Our results allow us to disentangle the competing complexities associated with nominal hole doping into components related to tolerance factor, A-site size variance, and concentration of JT active species that we capture as τ_JT_ in our model. The value of this model is that it could be used to develop a novel series of doped samples that sit on an isotherm with respect to *T*_LTO_, or in identifying anomalies in the crystal chemistry of other RPs n = 1 compounds based on their observed *T*_LTO_.

As our model system, we take La_2–*x*_Ba_*x*_CuO_4_, *x* = 0.125, which has been much studied on account of the anomalous suppression of superconducting *T*_C_ exhibited at the 1/8th doping level.^23^ On cooling, a high temperature tetragonal (HTT) to LTO transition at 254 K is followed by a first-order transition to a low temperature tetragonal (LTT) structure with an onset temperature of ca. 70 K that acts to suppress *T*_C_ toward 0 K. We synthesized a solid solution of formula La_1.875_Ba_0.125_(Cu_1–*y*_Mg_*y*_)_0.875_Cu_0.125_O_4_, in which JT-active Cu^2+^ is systematically substituted by JT-inactive Mg^2+^ (see Supporting Information (SI) for details), following similar procedures reported for La_1.85_Sr_0.15_Cu_0.7_Mg_0.3_O_4_.^24^ This particular substitution has the added benefit that the ionic radii of Cu^2+^ and Mg^2+^ are very similar (0.72 versus 0.73 Å).^2^

Figure 2(a) shows our high-resolution powder X-ray diffraction (PXRD) collected on 09A at the Taiwan Photon Source, and I11, Diamond Light Source, showing the LTO-to-HTT phase transition for La_1.875_Ba_0.125_(Cu_1–*y*_Mg_*y*_)_0.875_Cu_0.125_O_4_ with *y* = 0 and 0.5 (see SI for other compositions).

**Figure 2:**
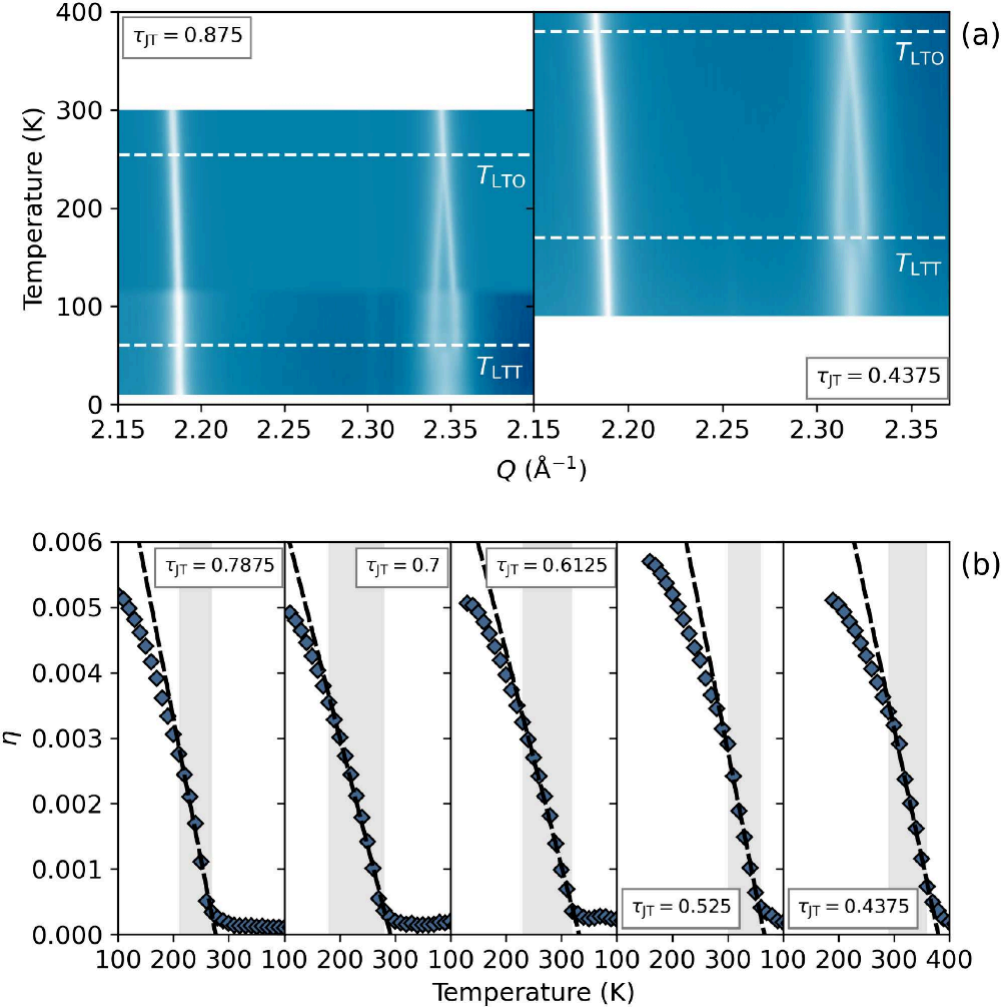
a) X-ray intensity heat map of the evolution of the (113) and (200)/(020) Bragg peaks of our experimental end-members of La_1.875_Ba_0.125_(Cu_1–*y*_Mg_*y*_)_0.875_Cu_0.125_O_4_ (*y* = 0 and *y* = 0.5). b) Temperature evolution of the lattice orthorhombicity (η) for the compositional series (*y* = 0.1–0.5 in 0.1 steps). τ_JT_ indicates the proportion of JT-active B–site cations. Regions over which the fit was performed are highlighted in gray.

The symmetry-breaking orthorhombic lattice strain provides a convenient way to precisely extract the transition temperatures, as shown in Figure 2(b). In Figure 3 we demonstrate a strong negative correlation between the fraction of JT-active Cu^2+^ in the samples (τ_JT_) and the LTO transition temperature (*T*_LTO:obs_). With the aid of high-resolution neutron powder diffraction data that enables us to determine the crystallographic positions of the oxygen atoms precisely, we calculate the Van Vleck Q_3_ mode—a signature for *d*_*z*^2^_ type orbital ordering (see SI). Figure 3 also shows a strong positive correlation between τ_JT_ and the Van Vleck Q_3_ mode and Figure 1(c)),^25^ suggesting that it is the underlying anisotropic nature of this control parameter that is the significant factor in controlling *T*_LTO:obs_. The Q_3_ mode in these structures is related to both the axial strain of the *ab* plane via the equatorial oxygen atoms that sit on special positions within the *I*4/*mmm* aristotype, and to the rumpling of the A-site cation and axial oxygen in the rock salt layer (Figure 1(c)). While the latter of these has been ascribed to be related to the specific pattern of octahedral rotations and tilts observed in selected RPs *n* = 2, A_3_B_2_O_7_ (A = Ca and Sr) compounds,^26^ it is not easy in practice to deconvolute which of these structural changes is more dominant in driving the change in *T*_LTO:obs_.

**Figure 3:**
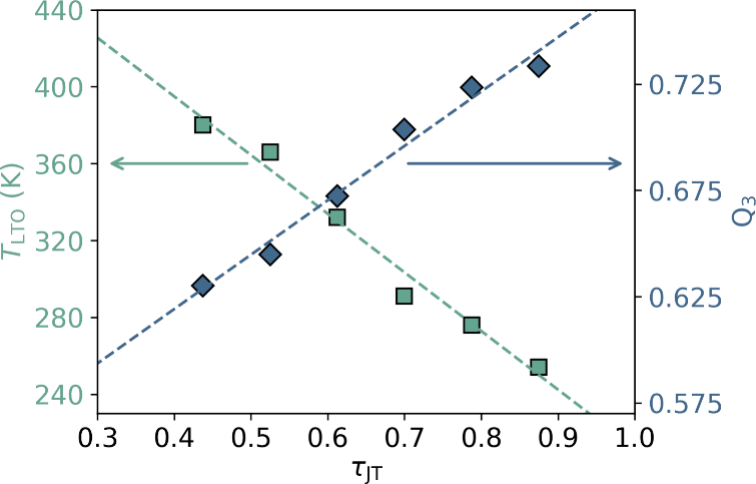
Trends in the LTO-to-HTT phase transition temperature, *T*_LTO_ (squares) and *Q*_3_ mode (diamonds) as a function of the concentration of JT-active species, τ_JT_ in Mg-substituted La_2–*x*_Ba_*x*_CuO_4_, *x* = 0.125.

In Figure 4(a), we put our results in the broader context of the hole-doped La_2–*x*_A_*x*_CuO_4_ cuprates, and show why the conventional approach of considering tolerance factor (τ) alone is insufficient to understanding the trend in *T*_LTO:obs_. Our starting point is to consider the effect of the change in τ, which is related to both the size of A-site substituents and the doping level (Cu^2+^ and Cu^3+^ having different radii), on *T*_LTO_. While for each series there is a strong correlation between τ and *T*_LTO_, τ clearly does not act as a universal predictor, as shown in Figure 4(a). Furthermore, in considering our Mg series, in which τ changes only a small amount, the trend appears to be reversed. Taking τ_JT_ on its own proves also to be an inadequate global predictor (Figure 4(b)). This suggests that the evolution of multiple control parameters must be considered simultaneously to determine a predictor for *T*_LTO_. Noting previous work that has also identified the effect of A-site variance *rσ*^2^ on transition temperatures,^33,34^ we fit simultaneously to 44 unique *T*_LTO:obs_ the model: *T*_LTO:calc_ = *a*(τ – τ_0_) + *bτ*_JT_ + *crσ*^2^, where *a* = −31418(949), τ_0_ = 0.89746(99), *b* = −404.1(40.0) and *c* = 35236(2427) are globally fit constants, determined in a least-squares fitting procedure (see SI). *a* and τ_0_ relate to the tolerance factor dependence of *T*_LTO:calc_, while *b* and *c* describe its linear dependence on τ_JT_ and *rσ*^2^, respectively. The quality of this fitting is shown in Figure 4(c), where the route mean squared deviation between *T*_LTO:obs_ and *T*_LTO:calc_ is only 13.3 K. Considering the varied chemistry on both A-site and B-sites, known oxygen nonstoichiometries in these materials, and the experimental resolution with which both composition and second-order phase transition temperatures are readily determined, the high level of agreement between experimental and predicted *T*_LTO_ is striking.

**Figure 4:**
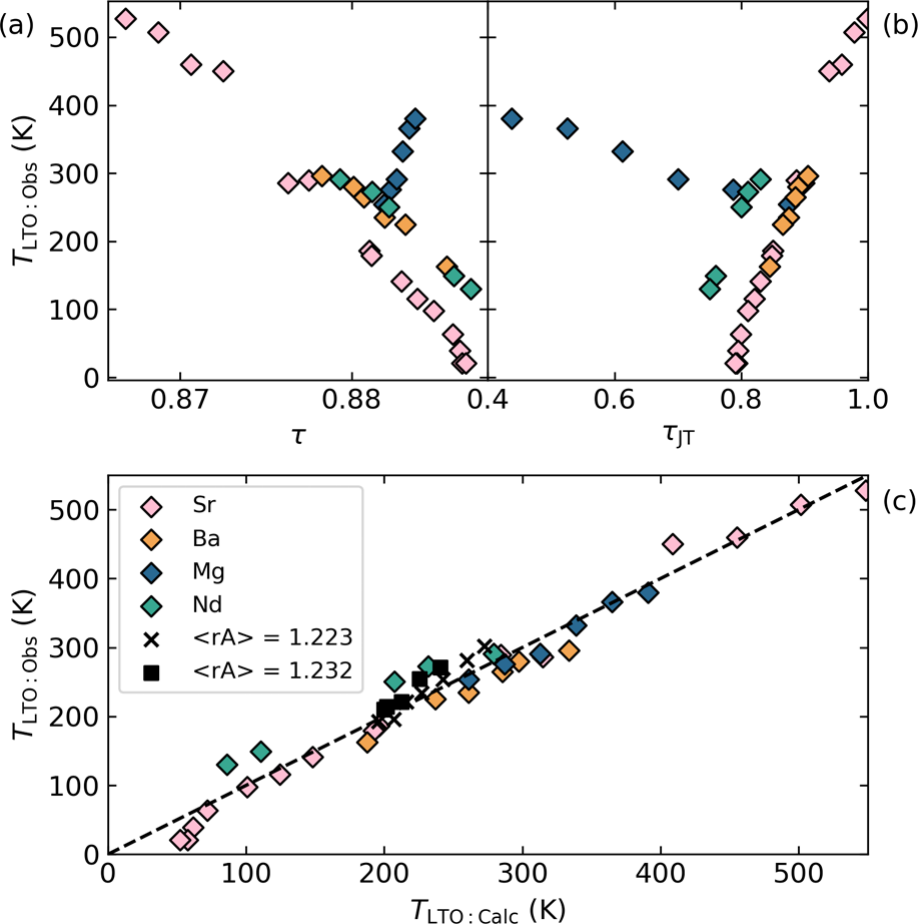
Experimentally observed *T*_LTO:obs_ for various cuprate series (La_2–*x*_Sr_*x*_CuO_4_ (Sr),^27−29^ La_2–*x*_Ba_*x*_CuO_4_ (Ba),^30^ La_1.875_Ba_0.125_(Cu_1–*y*_Mg_*y*_)_0.875_Cu_0.125_O_4_ (Mg), La_2–*x*_Nd_0.4_Sr_*x*_CuO_4_ (Nd)^31,32^ and variance^33^) indicated in the legend, and as discussed in the text. (a) *T*_LTO:obs_ versus τ, the calculated tolerance factor. (b) *T*_LTO:obs_ versus τ_JT_, the concentration of JT-species. (c) *T*_LTO:obs_ versus *T*_LTO:calc_ according to the model discussed in the text, with a *y* = *x* dashed line to guide the eye.

The availability of a model to accurately predict phase transition temperatures clearly has ramifications both for the curpate RP perovskites and more broadly on any perovskite-related materials with JT-active species. First, the cuprates are electronically very complex, and there will undoubtedly be regions of a phase diagram where there will be significant interplay between electron localization and structural distortions. Being able to identify such cases through deviations from the predicated *T*_LTO_ is clearly important. Second, there is a strong interplay between the observed structural symmetry and the occurrence of charge density wave order that acts to suppress superconductivity in these and related systems.^23,35^ Results are typically viewed on a phase diagram with the independent variable *x* taken to be hole doping, which is also equivalent to τ_JT_ in the case of the parent compound La_2_CuO_4_. Since our results show that the LTO–HTT phase boundary varies in a nontrivial manner, an alternative approach of studying electronic phenomena in the cuprates might be to construct a series of samples that vary in composition such that they sit on an isotherm with respect to the predicted *T*_LTO:calc_, thus deconvoluting the effects of changing structural degrees of freedom and symmetry on the phase diagram. By assuming that doping level (*x* or τ_JT_), τ and *rσ*^2^ may be treated as independent variables, we construct isotherms at *T*_LTO_ = 600, 300 and 100 K (Figure 5 and the SI). For the synthetic chemist, Figure 5 should be interpreted as a guide for constructing a series of samples with a fixed *T*_LTO_ across a desired doping regime by varying τ and *rσ*^2^ as necessary. While some chemical space may prove inaccessible, we note recent reports of ultrahigh variance high-pressure perovskites Ba_0.5_Ce_0.5_MnO_3_ with average A-site valence and ionic radii near to that of La^3+^ and an *rσ*^2^ in excess of 0.05 Å^2^.^36^ This suggests that with some synthetic endeavor it may be possible to stay within a given isotherm across a broad range of dopings (*x*). For example, a series of high-variance hole-doped (x) compounds (La_1–*y*_(Ba_0.5_Ce_0.5_)_*y*_)_2–*x*_Sr_*x*_CuO_4_ can lie on the 600 K isotherm.

**Figure 5:**
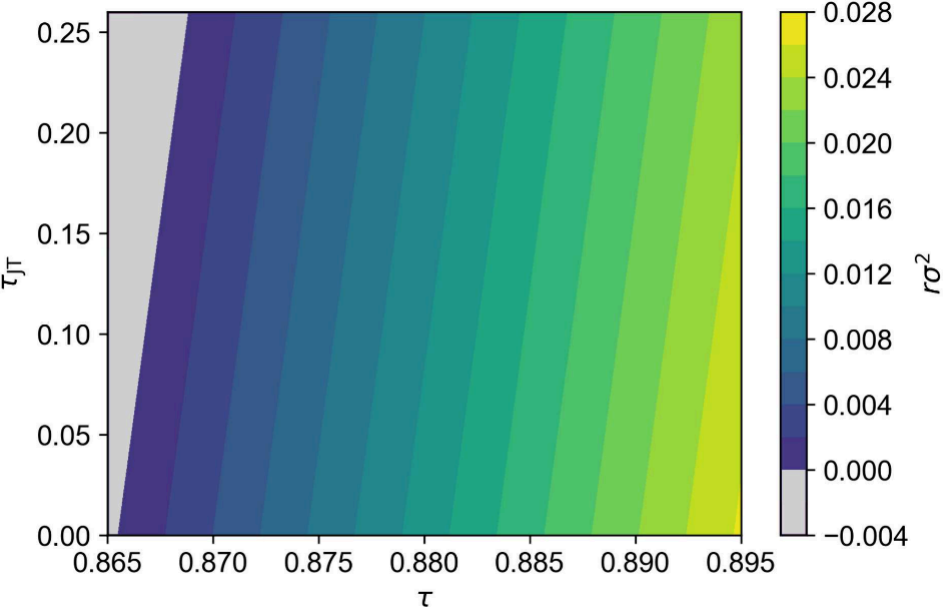
Isotherms *T*_LTO_ = 600 K computed as a function of tolerance factor (τ), doping level (τ_JT_), and A-site variance (*rσ*^2^). Physically inaccessible regions on account of negative A-site variances are shaded in gray.

Finally, with the resurgent interest in the nickelate RP perovskites due to the reported high-pressure superconductivity,^37,38^ it is perhaps timely to reinvestigate the displacive phase transitions in these materials. Indeed, La_2_NiO_4_ is reported to have an HTT-to-LTO phase transition below 770 K.^39^ In principle one can calculate an expected transition temperature based on our model with τ = 0.883, τ_JT_ and *rσ*^2^ = 0 (using Shannon ionic radii^2^ for Ni^2+^ of 0.69 Å), which gives *T*_LTO:calc_ = 442(34) K, which is in poor agreement with the reported *T*_LTO:obs_. However, a closer investigation of the crystal structure of La_2_NiO_4_ at 1073 K (i.e., well above *T*_LTO_) suggests an ionic radius for Ni^2+^ of 0.716 Å (see SI for our justification); τ = 0.873 is more appropriate. Taking this updated value gives *T*_LTO:calc_ = 780(39) K, in excellent agreement with the experimental temperature. The question remains why the tabulated ionic radii for six-coordinate Ni^2+^ appear at odds with that observed experimentally in La_2_NiO_4_, a point that warrants further investigation and may be related to the strong hybridization between oxygen 2*p* and Ni 3*d* states in these materials,^40^ leading to anisotropic bonding. In general, it is hoped that our work will help identify outliers to the proposed model for *T*_LTO:calc_, prompting more detailed investigations into effects like those discussed above, and ones related to oxygen nonstoichiometry, that are prevalent in many highly correlated systems with variable oxidation states, but often poorly characterized

In conclusion, our investigation of structural phase transitions in a series of cuprate RP oxides has identified the effect that single-ion anisotropy, associated with the concentration of JT-active sites, has on *T*_LTO_. Considering this effect and those already known to be associated with perovskites (tolerance factor and A-site size variance), we have fitted a linear model to 44 various compositions of doped RP n = 1 cuprates, with a route mean squared error between calculated and observed transition temperature of only 13.3 K. The utility of the predicted model is illustrated both in terms of how it might be used to construct a series of doped samples that form an isotherm with respect to their structural phase transition temperature and in identifying potential anomalies in the literature in the broader family of RP compounds that are of the utmost relevance in the current search for high-temperature superconductivity in the nickelates. Furthermore, our approach might be used to model phase transitions in n = 2 RPs that display similar octahedral tilting transitions, a large number of which contain JT-active cations.

## Data Availability

The data underpinning the fit to the phase transition temperatures (https://doi.org/10.6084/m9.figshare.28174682.v1) and the raw XRD data (https://doi.org/10.6084/m9.figshare.28173971.v1), are available at the given links.

## Supporting Information Available

Detailed synthesis, Rietveld refinements and associated crystallographic information. (PDF)
