# Supplementary material for: Interplay between Jahn–Teller Distortions and Structural Phase Transitions in Ruddlesden–Poppers
Source: J Am Chem Soc. 2025 Feb 18;147(9):7209–13. doi: 10.1021/jacs.5c00459 (PMC11887055; doi:10.1021/jacs.5c00459)
Supplement: Supplementary file 1 — ja5c00459_si_001.pdf [file ja5c00459_si_001.pdf]

Interplay between Jahn-Teller distortions and structural phase transitions in Ruddlesden-Poppers

Anna Herlihy,[1] Wei-Tin Chen,[2,3] Clemens Ritter,[4] Yu-Chun Chuang,[5,6] Mark S. Senn,[7,*]
[1]Diamond Light Source, Harwell Campus, Oxfordshire, OX11 0DE, U.K.
[2]Center for Condensed Matter Sciences and Center of Atomic Initiative for New Materials, National Taiwan University, Taipei 10617, Taiwan
[3]Taiwan Consortium of Emergent Crystalline Materials, Ministry of Science and Technology, Taipei 10622, Taiwan
[4]Institut Laue-Langevin, 71 Avenue des Martyrs, CS20156, 38042 Grenoble Cédex 9, France
[5]National Synchrotron Radiation Research Center, Hsinchu, 300092, Taiwan
[6]Department of Chemistry, National Taiwan University, Taipei 10617, Taiwan
[7]Department of Chemistry, University of Warwick, Gibbet Hill, Coventry, CV4 7AL, U.K.

Supplementary Information

1. Solid-state synthesis of Mg-substituted $La_{1.875}Ba_{0.125}CuO_4$

$La_{1.875}Ba_{0.125}(Cu_{1-y}Mg_y)_{0.875}Cu_{0.125}O_4$ (y=0-0.5 in 0.1 steps) was synthesised by mixing predried La_2O_3 (99.99%, Sigma Aldrich), $BaCO_3$ (99.95%, Alfa Aesar), CuO (99.999%, Sigma Aldrich) and MgO (99.99%, Sigma Aldrich) in the stoichiometric ratios detailed in Table 1.1. Note that a stoichiometric ratio of 0.125 of the Cu is 3+ and is not replaced by the Mg^{2+} cations.

Reactant	Ratio
La_2O_3	0.9375
$BaCO_3$	0.125
CuO	0.875(1-y)+0.125
MgO	0.875y

Table 1.1: Reactants and their stoichiometric y-dependent ratios.

Powders were ground into a homogeneous mixture and then pressed into pellets using a Specac 10 tonne die press. Pellets were placed into a zirconia crucible and calcined in air at 900°C for 20 hours. Three heating cycles were then carried out at 1050°C for 15 hours, 110°C for 20 hours and 1200°C for 20 hours, with pellets cooled to room temperature, ground and pre-pressed between each cycle.

2. Data collection – Variable-temperature XRD

Variable-temperature powder diffraction patterns of the $La_{1.875}Ba_{0.125}(Cu_{1-y}Mg_y)_{0.875}Cu_{0.125}O_4$ series were measured using high-resolution XRD beamlines at Diamond Light Source (DLS) and Taiwan Photon Source (TPS), with different experimental details for each sample. All variable temperature measurements were performed on warming. All data were collected using a MYTHEN detector. All wavelengths and instrument-dependent parameters (peak shapes and axial divergence) were determined via Rietveld refinements of a Si NIST standard.

y = 0.0

Data were obtained from 100–300 K in 10 K steps, also using beamline 09A, with an energy of 20 keV (0.61990 Å). The sample was loaded into a 0.1 mm diameter Lindemann glass capillary and the temperature was controlled using an Oxford Cryostream 800+ with a ramp rate of 2.5 K/min. High temperature data were collected from 300–400 K in steps of 10 K using beamline I11 (DLS).

An energy of 15 keV (0.82686 Å) was used and an Oxford Cryostream+ used to control the temperature with a ramp rate of 6 K/min.

y = 0.1

Data were collected using beamline I11 (DLS) with an energy of 15 keV (0.82686 Å). The sample was loaded into a 0.3 mm diameter borosilicate glass capillary and data were collected from 100–400 K in 10 K steps. The temperature was controlled using an Oxford Cryostream Plus.

y = 0.2, 0.3, 0.4 and 0.5

Data were collected from 100–400 K in steps of 10 K using beamline 09A. The samples were loaded into 0.1 mm Lindemann glass capillaries. An energy of 12 keV (1.03342 Å) was used and an Oxford Cryostream800+ used to control the temperature with a ramp rate of 2.5 K/min.

Neutron diffraction (ambient)

Powder neutron diffraction (PND) data were collected for $La_{1.875}Ba_{0.125}(Cu_{1-y}Mg_{y})_{0.875}Cu_{0.125}O_4$ (y=0, 0.3, and 0.5) on the high-resolution diffractometer, D2B at the Institut Laue Langevin (ILL), with λ = 1.594983 Å. The samples were loaded into 9 mm cylindrical vanadium cans and high-resolution data was collected for 2 hours per sample at ambient temperature.

3. Refinement Protocol

Structure modelling for Rietveld refinements against the variable-temperature XRD data and small-box modelling of X-ray PDFs was carried out using the approach outlined below using TOPAS Academic v6 (Coelho, 2018).

A symmetry-adapted approach was used whereby for LTO and HTT phases, refinements were performed using a common subgroup, $Pccn$ with appropriate symmetry constraints on atom displacements and lattice parameters. This means that all phases were modelled using the supercell structure so that refined parameters of the HTT structure—which conventionally would be described by a smaller unit cell—can be directly compared. The OP associated with the octahedral rotation is described by the irrep. X_3^+ with an OPD (a, b). For the HTT phase, the OPD is (0,0)—where there is no rotation in the average structure and for the LTO phase, the OPD is (a, 0). A second distortion is active within the HTT and LTO—the Γ_1^+ mode (with OPD (a))—which describes a symmetry-conserving distortion where La/Ba and apical O atoms displace with a combined stretching motion along the c-axis.

Irrep / mode	La/Ba	O1	O2
X_3^+	1	-3.0232	1.3905
Γ_1^+	1	-1.1026	n/a

Table 3.1: Ratio between the La/Ba, O1 (apical) and O2 (equatorial) distortion modes described by the X_3^+ and Γ_1^+ irreps.

In each model, two distortion parameters were refined, one describing the La/Ba X_3^+ distortion, and another describing the La/Ba Γ_1^+ distortion. All other modes transforming as the same irrep, acting on the oxygen atoms, were fixed to a ratio which was based on values determined from

previous, unpublished single crystal studies (carried out within the Senn group) of isostructural $La_{1.675}Eu_{0.2}Sr_{0.125}CuO_4$ (LESCO) and reported in Table 3.1. See file "example_refinement_file_for_LBMCO.inp" for exact relationships between mode ratios and distortions. These values were validated by performing unconstrained refinements against neutron powder diffraction data of $La_{1.875}Ba_{0.125}(Cu_{1-y}Mg_y)_{0.875}Cu_{0.125}O_4$ (y=0, 0.3, and 0.5) (see file example_neutron_refinement.inp). The X_3^+ distortion acts on two distinct oxygen positions; the apical oxygen denoted 'O1', and the equatorial oxygen, denoted 'O2', whereas the Γ_1^+ distortion acts only on the apical O1 oxygen position.

Constraining oxygen distortions to the La distortions was vital for maintaining the octahedral geometry since freely refined modes resulted in incorrect coupling of the individual components due to the insensitivity of X-rays to the lighter oxygen atoms. The reported X_3^+ mode amplitudes are the parent-cell-normalized amplitudes (AP values) defined by ISODISTORT (Campbell et al, 2006).

The atomic displacement parameters of the equatorial oxygen atoms were constrained to be equal, regardless of the phase symmetry and those describing the apical oxygen atoms were constrained to be 1.8× of the equatorial oxygen atoms (again, informed by single crystal LESCO data). Atomic displacement parameters for the remaining atom site types (A and B site atoms) were allowed to refine freely with no further constraints. Modelling of anisotropic peak broadening was carried out using the symmetry-dependent Stephens strain model (Stephens, 1999) for each phase in all sample.

4. Structural parameters

The Q_3 mode describes the Jahn-Teller instability related d_{z^2}-type orbital ordering which is present in the low temperature orthorhombic and high temperature tetragonal phases of LBCO:

$$Q_3 = \frac{2(l-s)}{\sqrt{2}},$$

where l and s refer to long and short B-O bond lengths respectively (van Vleck, 1939).

The 'orthorhombicity' parameter, equivalent to orthorhombic strain, η,

$$\eta = \left|\frac{2(a-b)}{(a+b)}\right|,$$

was calculated from refined lattice parameters of the $La_{1.875}Ba_{0.125}(Cu_{1-y}Mg_y)_{0.875}Cu_{0.125}O_4$ structures. An increasingly large value corresponds to a more orthorhombic structure and a value of zero corresponds to a tetragonal structure.

In order to determine the phase transition temperature, T_c more precisely than otherwise allowed by the minimum temperature step used in this experiment (10 K), Landau theory was used (Dove, 1997). We use the secondary order parameter, orthorhombic strain (η) as defined above, which can be approximated by the relationship;

$$\eta(T) = \eta_{t=0}\left[\frac{T_C - T}{T_C}\right]$$

Least squares fitting was performed using TOPAS over a chosen finite range tabulated below, close to the apparent phase transition temperature, in accordance with Landau theory.

Composition (*y*)	Temperature range for fitting (K)
0.1	210-270
0.2	180-280
0.3	230-320
0.4	300-360
0.5	290-360

Table 4.1: Temperature ranges used for linear least squares fitting.

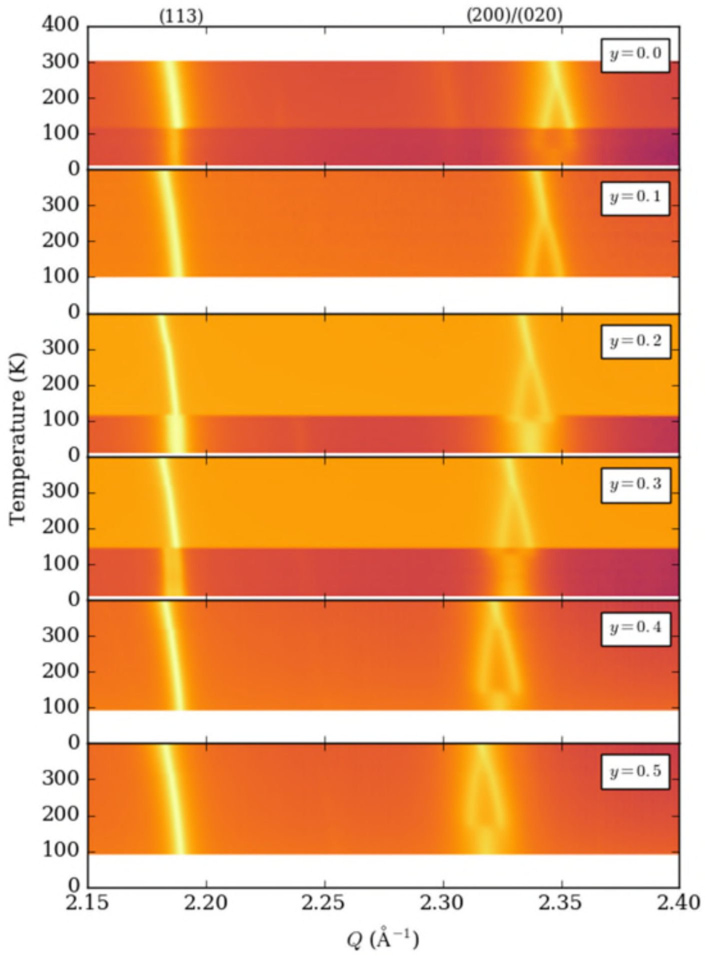

Figure 4.1: An X-ray intensity heat map of the evolution of the (113) and (200)/(020) Bragg peaks of $La_{1.875}Ba_{0.125}(Cu_{1-y}Mg_y)_{0.875}Cu_{0.125}O_4$ with temperature. White sections reflect temperatures at which data were not collected and changes in contrast between low and high temperatures are a result of different scattering intensities between data collected from different experiments.

Temperature (K)	Phase	R_{wp}	a (Å)	b (Å)	c (Å)	X_3^+	Γ_1^+	$B_{iso}(A)$ (Å^2)	$B_{iso}(B)$ (Å^2)	$B_{iso}(O)$ (Å^2)
100	LTO	3.83998	5.34991(3)	5.37775(3)	13.18482(6)	0.076(5)	0.008(1)	0.917(8)	0.80(2)	1.70(5)
110	LTO	3.81494	5.35018(3)	5.37765(3)	13.18572(6)	0.075(5)	0.008(1)	0.928(8)	0.82(2)	1.75(5)
120	LTO	3.81601	5.35065(3)	5.37736(3)	13.18708(6)	0.078(5)	0.008(1)	0.936(8)	0.83(2)	1.70(5)
130	LTO	3.84731	5.35122(3)	5.37704(3)	13.18859(6)	0.077(5)	0.008(1)	0.953(8)	0.82(2)	1.67(5)
140	LTO	3.81961	5.35186(2)	5.37662(3)	13.19015(6)	0.072(6)	0.008(1)	0.971(8)	0.85(2)	1.70(5)
150	LTO	3.80601	5.35252(2)	5.37614(3)	13.19182(6)	0.072(6)	0.009(1)	0.995(8)	0.87(2)	1.67(5)
160	LTO	3.79134	5.35330(2)	5.37564(3)	13.19364(6)	0.078(5)	0.009(1)	1.006(8)	0.87(2)	1.73(5)
170	LTO	3.78507	5.35408(2)	5.37509(3)	13.19555(6)	0.077(5)	0.009(1)	1.020(8)	0.89(2)	1.68(5)
180	LTO	3.82853	5.35504(2)	5.37443(3)	13.19787(6)	0.075(6)	0.009(1)	1.040(8)	0.90(2)	1.61(5)
190	LTO	3.83692	5.35590(2)	5.37381(3)	13.19978(6)	0.075(6)	0.008(1)	1.048(8)	0.93(2)	1.60(5)
200	LTO	3.89863	5.35679(2)	5.37320(3)	13.20192(6)	0.065(7)	0.008(1)	1.063(8)	0.95(2)	1.69(5)
210	LTO	3.88782	5.35769(2)	5.37250(3)	13.20398(6)	0.063(7)	0.009(1)	1.078(8)	0.94(2)	1.71(5)
220	LTO	3.87393	5.35869(3)	5.37180(3)	13.20618(6)	0.059(7)	0.009(1)	1.095(8)	0.96(2)	1.69(5)
230	LTO	3.86662	5.35971(3)	5.37098(3)	13.20834(6)	0.059(7)	0.010(1)	1.101(8)	0.98(2)	1.66(5)
240	LTO	3.83101	5.36095(3)	5.37003(3)	13.21058(6)	0.053(9)	0.010(1)	1.109(8)	1.00(2)	1.66(5)
250	LTO	3.77937	5.36262(4)	5.36856(5)	13.21295(6)	0.058(8)	0.009(1)	1.102(8)	1.01(2)	1.69(5)
260	LTO	3.77786	5.36434(4)	5.36708(6)	13.21593(5)	0.066(8)	0.009(1)	1.111(8)	0.99(2)	1.65(5)
270	LTO	3.96457	5.36497(3)	5.36681(5)	13.21868(5)	0.075(7)	0.009(1)	1.103(8)	1.02(2)	1.62(5)
280	HTT	3.65581	5.36585(2)	5.36585(2)	13.22079(5)	0	0.010(1)	1.093(7)	0.98(2)	1.57(5)
290	HTT	3.65132	5.36626(2)	5.36626(2)	13.22284(5)	0	0.010(1)	1.096(7)	0.99(2)	1.53(5)
300	HTT	3.65079	5.36673(2)	5.36673(2)	13.22485(5)	0	0.009(1)	1.106(7)	1.01(2)	1.48(5)
310	HTT	3.62892	5.36723(2)	5.36723(2)	13.22675(5)	0	0.009(1)	1.120(7)	1.03(2)	1.48(4)
320	HTT	3.57512	5.36785(2)	5.36785(2)	13.22897(5)	0	0.009(1)	1.144(7)	1.05(2)	1.48(4)
330	HTT	3.60436	5.36831(2)	5.36831(2)	13.23054(5)	0	0.010(1)	1.154(7)	1.05(2)	1.49(4)
340	HTT	3.55584	5.36897(2)	5.36897(2)	13.23269(5)	0	0.010(1)	1.171(7)	1.07(2)	1.50(4)
350	HTT	3.56797	5.36957(2)	5.36957(2)	13.23458(5)	0	0.009(1)	1.175(7)	1.07(2)	1.50(4)
360	HTT	3.58281	5.37021(2)	5.37021(2)	13.23644(5)	0	0.008(1)	1.193(7)	1.07(2)	1.48(4)
370	HTT	3.59378	5.37080(2)	5.37080(2)	13.23830(5)	0	0.010(1)	1.201(7)	1.10(2)	1.45(4)

380	HTT	3.5853	5.37141(2)	5.37141(2)	13.24011(5)	0	0.011(1)	1.222(7)	1.09(2)	1.45(4)
390	HTT	3.59704	5.37204(2)	5.37204(2)	13.24195(5)	0	0.011(1)	1.230(7)	1.12(2)	1.47(4)
400	HTT	3.57683	5.37266(2)	5.37266(2)	13.24375(5)	0	0.011(1)	1.251(7)	1.13(2)	1.49(4)

Table 4.2: Refined parameters from Rietveld refinements against variable temperature XRD patterns of $La_{1.875}Ba_{0.125}(Cu_{1-y}Mg_y)_{0.875}Cu_{0.125}O_4$ y = 0.1.

Temperature (K)	Phase	R_{wp}	a (Å)	b (Å)	c (Å)	X_3^+	Γ_1^+	$B_{iso}(A)$ (Å^2)	$B_{iso}(B)$ (Å^2)	$B_{iso}(O)$ (Å^2)
110	LTO	3.01603	5.36178(1)	5.38819(1)	13.15081(3)	0.079(2)	0.0014(7)	0.483(4)	0.22(1)	0.59(2)
120	LTO	3.02293	5.36222(1)	5.38803(1)	13.15224(3)	0.079(2)	0.0018(7)	0.495(4)	0.23(1)	0.59(2)
130	LTO	3.02242	5.36275(1)	5.38771(1)	13.15383(3)	0.078(2)	0.0020(7)	0.504(4)	0.24(1)	0.58(2)
140	LTO	3.02696	5.36336(1)	5.38734(1)	13.15548(3)	0.076(2)	0.0021(7)	0.517(4)	0.25(1)	0.58(2)
150	LTO	3.04334	5.36410(1)	5.38697(1)	13.15715(3)	0.073(3)	0.0022(7)	0.530(4)	0.26(1)	0.58(2)
160	LTO	3.02729	5.36480(1)	5.38648(1)	13.15897(3)	0.070(3)	0.0022(7)	0.541(4)	0.27(1)	0.59(2)
170	LTO	3.02838	5.36559(1)	5.38600(1)	13.16085(3)	0.067(3)	0.0021(7)	0.553(4)	0.28(1)	0.60(2)
180	LTO	3.03291	5.36640(1)	5.38546(1)	13.16279(3)	0.064(3)	0.0016(7)	0.567(4)	0.29(1)	0.61(2)
190	LTO	3.03561	5.36724(1)	5.38490(1)	13.16485(3)	0.062(3)	0.0019(7)	0.577(4)	0.30(1)	0.60(2)
200	LTO	3.04432	5.36812(1)	5.38432(1)	13.16692(3)	0.058(3)	0.0016(7)	0.590(4)	0.31(1)	0.61(2)
210	LTO	3.05913	5.36904(1)	5.38371(1)	13.16904(3)	0.055(3)	0.0013(7)	0.603(4)	0.31(1)	0.62(2)
220	LTO	3.07525	5.36998(1)	5.38309(1)	13.17121(3)	0.052(3)	0.0011(7)	0.614(4)	0.31(1)	0.63(2)
230	LTO	3.07324	5.37095(1)	5.38236(1)	13.17348(3)	0.048(4)	0.0012(7)	0.626(4)	0.31(1)	0.66(2)
240	LTO	3.07141	5.37201(1)	5.38164(1)	13.17566(3)	0.045(4)	0.0013(7)	0.638(4)	0.31(1)	0.68(2)
250	LTO	3.07742	5.37319(1)	5.38082(1)	13.17785(3)	0.038(5)	0.0014(7)	0.648(4)	0.30(1)	0.69(2)
260	LTO	3.08235	5.37439(2)	5.37983(2)	13.17986(3)	0.034(6)	0.0018(7)	0.654(3)	0.29(1)	0.70(2)
270	LTO	3.22588	5.37572(2)	5.37868(3)	13.18284(3)	0.03(7)	0.0019(7)	0.657(4)	0.30(1)	0.74(2)
280	LTO	3.28001	5.37645(2)	5.37840(3)	13.18533(3)	0.027(8)	0.0016(7)	0.671(4)	0.31(1)	0.78(2)
290	LTO	3.27113	5.37698(2)	5.37840(3)	13.18720(3)	0.027(8)	0.0008(7)	0.680(4)	0.31(1)	0.82(2)
300	HTT	3.2703	5.37803(1)	5.37803(1)	13.18950(2)	0	0.0012(7)	0.692(3)	0.310(10)	0.84(2)
310	HTT	3.25503	5.37848(1)	5.37848(1)	13.19112(2)	0	0.0009(7)	0.706(3)	0.314(10)	0.85(2)
320	HTT	3.23028	5.37967(1)	5.37967(1)	13.19523(2)	0	0.0002(7)	0.725(3)	0.322(10)	0.91(2)

330	HTT	3.23502	5.38033(1)	5.38033(1)	13.19725(2)	0	0.0008(7)	0.740(3)	0.335(10)	0.98(2)
340	HTT	3.19404	5.38095(1)	5.38095(1)	13.19936(2)	0	0.0002(7)	0.753(3)	0.358(10)	1.04(2)
350	HTT	3.18783	5.38161(1)	5.38161(1)	13.20134(2)	0	0.0001(7)	0.771(3)	0.375(10)	1.11(2)
360	HTT	3.15229	5.38228(1)	5.38228(1)	13.20330(2)	0	0.0004(7)	0.783(3)	0.376(9)	1.17(2)
370	HTT	3.13272	5.38295(1)	5.38295(1)	13.20525(2)	0	0.0015(7)	0.792(3)	0.402(10)	1.18(2)
380	HTT	3.17784	5.38365(1)	5.38365(1)	13.20711(2)	0	0.002(07)	0.803(3)	0.412(10)	1.23(2)
390	HTT	3.13873	5.38435(1)	5.38435(1)	13.20914(2)	0	0.0026(7)	0.806(3)	0.437(10)	1.28(2)
400	HTT	3.19015	5.38508(1)	5.38508(1)	13.21102(2)	0	0.0023(7)	0.813(3)	0.451(10)	1.29(2)

Table 4.3: Refined parameters from Rietveld refinements against variable temperature XRD patterns of $La_{1.875}Ba_{0.125}(Cu_{1-y}Mg_y)_{0.875}Cu_{0.125}O_4$ y = 0.2.

Temperature (K)	Phase	R_{wp}	a (Å)	b (Å)	c (Å)	X_3^+	Γ_1^+	$B_{iso}(A)$ (Å^2)	$B_{iso}(B)$ (Å^2)	$B_{iso}(O)$ (Å^2)
130	LTO	2.65516	5.37690(1)	5.40418(1)	13.09606(3)	0.093(2)	0.0200(6)	1.021(3)	0.92(1)	1.41(2)
140	LTO	2.65524	5.37716(1)	5.40428(1)	13.09763(3)	0.096(2)	0.0197(6)	1.022(3)	0.93(1)	1.41(2)
150	LTO	2.59661	5.37755(1)	5.40408(1)	13.09949(3)	0.096(2)	0.0179(6)	1.042(3)	0.92(1)	1.45(2)
160	LTO	2.58097	5.37811(1)	5.40387(1)	13.10126(3)	0.091(2)	0.0144(5)	1.063(3)	0.91(1)	1.51(2)
170	LTO	2.56584	5.37872(1)	5.40353(1)	13.10322(3)	0.087(2)	0.0147(5)	1.093(3)	0.93(1)	1.58(2)
180	LTO	2.55542	5.37940(1)	5.40312(1)	13.10524(2)	0.085(2)	0.0151(5)	1.123(3)	0.975(9)	1.56(2)
190	LTO	2.55851	5.38014(1)	5.40273(1)	13.10722(2)	0.086(2)	0.0151(5)	1.141(3)	0.956(9)	1.56(2)
200	LTO	2.55439	5.38088(1)	5.40227(1)	13.10933(2)	0.086(2)	0.0154(5)	1.151(3)	0.964(9)	1.53(2)
210	LTO	2.52885	5.38168(1)	5.40183(1)	13.11142(2)	0.085(2)	0.0170(5)	1.160(3)	0.978(9)	1.54(2)
220	LTO	2.55959	5.38252(1)	5.40137(1)	13.11346(2)	0.083(2)	0.0170(5)	1.169(3)	0.987(9)	1.57(2)
230	LTO	2.59245	5.38336(1)	5.40085(1)	13.11567(2)	0.079(2)	0.0148(5)	1.181(3)	0.997(9)	1.63(2)
240	LTO	2.6634	5.38426(1)	5.40035(1)	13.11785(3)	0.077(2)	0.0169(6)	1.197(3)	1.00(1)	1.57(2)
250	LTO	2.67144	5.38515(1)	5.39975(1)	13.12025(3)	0.075(2)	0.0190(6)	1.218(3)	0.99(1)	1.52(2)
260	LTO	2.72223	5.38610(1)	5.39916(1)	13.12252(3)	0.077(2)	0.0217(6)	1.231(3)	0.99(1)	1.52(2)
270	LTO	2.6143	5.38708(1)	5.39847(1)	13.12492(2)	0.073(2)	0.0217(5)	1.231(3)	1.023(9)	1.52(2)
280	LTO	2.5217	5.38806(1)	5.39783(1)	13.12709(2)	0.072(2)	0.0241(5)	1.231(3)	1.030(9)	1.59(2)
290	LTO	2.53784	5.38942(1)	5.39692(1)	13.12970(2)	0.069(2)	0.0215(5)	1.236(3)	1.028(9)	1.62(2)

300	LTO	2.61058	5.39063(1)	5.39594(2)	13.13242(2)	0.064(3)	0.0201(5)	1.255(3)	1.054(9)	1.58(2)
310	LTO	2.7991	5.39157(1)	5.39531(2)	13.13517(3)	0.061(3)	0.0219(6)	1.264(3)	1.03(1)	1.50(2)
320	LTO	2.78957	5.39307(2)	5.39497(2)	13.14032(2)	0.056(3)	0.0190(6)	1.307(3)	1.10(1)	1.50(2)
330	LTO	2.84251	5.39365(2)	5.39538(2)	13.14233(2)	0.057(3)	0.0137(6)	1.335(3)	1.14(1)	1.65(2)
340	HTT	2.78602	5.39481(1)	5.39481(1)	13.14462(2)	0	0.0128(6)	1.339(3)	1.14(1)	1.78(2)
350	HTT	2.69071	5.39540(1)	5.39540(1)	13.14687(2)	0	0.0118(6)	1.369(3)	1.133(9)	1.78(2)
360	HTT	2.7226	5.39602(1)	5.39602(1)	13.14899(2)	0	0.0051(6)	1.399(3)	1.155(9)	1.99(2)
370	HTT	2.74573	5.39669(1)	5.39669(1)	13.15094(2)	0	0.0068(6)	1.418(3)	1.17(1)	1.68(2)
380	HTT	2.71254	5.39733(1)	5.39733(1)	13.15297(2)	0	0.0069(6)	1.439(3)	1.179(9)	1.99(2)
390	HTT	2.68224	5.39792(1)	5.39792(1)	13.15479(2)	0	0.0053(6)	1.457(3)	1.222(9)	2.00(2)
400	HTT	2.72633	5.39863(1)	5.39863(1)	13.15668(2)	0	0.0053(6)	1.486(3)	1.24(1)	2.00(2)

Table 4.4: Refined parameters from Rietveld refinements against variable temperature XRD patterns of $La_{1.875}Ba_{0.125}(Cu_{1-y}Mg_y)_{0.875}Cu_{0.125}O_4$ y = 0.3.

Temperature (K)	Phase	R_{wp}	a (Å)	b (Å)	c (Å)	X_3^+	Γ_1^+	$B_{iso}(A)$ (Å^2)	$B_{iso}(B)$ (Å^2)	$B_{iso}(O)$ (Å^2)
160	LTO	2.40568	5.39145(1)	5.42227(1)	13.03912(3)	0.097(2)	0.0290(5)	0.663(3)	0.651(10)	1.09(2)
170	LTO	2.34336	5.39174(1)	5.42221(1)	13.04116(3)	0.098(2)	0.0290(5)	0.677(3)	0.663(9)	1.09(2)
180	LTO	2.32485	5.39222(1)	5.42204(1)	13.04319(3)	0.097(2)	0.0286(5)	0.690(3)	0.678(9)	1.08(2)
190	LTO	2.32966	5.39279(1)	5.42182(1)	13.04517(3)	0.095(2)	0.0282(5)	0.704(3)	0.684(9)	1.09(2)
200	LTO	2.35117	5.39343(1)	5.42155(1)	13.04718(3)	0.094(2)	0.0285(5)	0.717(3)	0.705(10)	1.10(2)
210	LTO	2.3352	5.39410(1)	5.42120(1)	13.04934(3)	0.093(2)	0.0285(5)	0.729(3)	0.714(9)	1.10(2)
220	LTO	2.33614	5.39482(1)	5.42084(1)	13.05148(3)	0.092(2)	0.0288(5)	0.741(3)	0.722(10)	1.13(2)
230	LTO	2.33095	5.39557(1)	5.42045(1)	13.05368(3)	0.090(2)	0.0288(5)	0.756(3)	0.728(9)	1.16(2)
240	LTO	2.32928	5.39635(1)	5.42004(1)	13.05590(3)	0.088(2)	0.0288(5)	0.772(3)	0.741(10)	1.17(2)
250	LTO	2.35161	5.39704(1)	5.41969(1)	13.05777(3)	0.085(2)	0.0287(5)	0.787(3)	0.753(10)	1.18(2)
260	LTO	2.31731	5.39796(1)	5.41912(1)	13.06047(3)	0.082(2)	0.0287(5)	0.805(3)	0.770(10)	1.20(2)
270	LTO	2.29728	5.39879(1)	5.41858(1)	13.06295(3)	0.079(2)	0.0288(5)	0.823(3)	0.794(9)	1.21(2)
280	LTO	2.32523	5.39954(1)	5.41819(1)	13.06490(3)	0.075(2)	0.0283(5)	0.839(3)	0.803(10)	1.24(2)
290	LTO	2.3421	5.40056(1)	5.41757(1)	13.06767(3)	0.073(2)	0.0283(5)	0.857(3)	0.819(10)	1.29(2)

300	LTO	2.37484	5.40137(1)	5.41712(1)	13.06974(3)	0.068(2)	0.0279(5)	0.871(3)	0.824(10)	1.35(2)
310	LTO	2.40682	5.40187(1)	5.41683(1)	13.07103(3)	0.065(3)	0.0276(5)	0.880(3)	0.821(10)	1.38(2)
320	LTO	2.37907	5.40472(1)	5.41492(1)	13.07820(3)	0.052(3)	0.0249(5)	0.920(3)	0.823(10)	1.41(2)
330	LTO	2.37695	5.40601(1)	5.41404(2)	13.08082(3)	0.045(4)	0.0254(5)	0.930(3)	0.837(10)	1.41(2)
340	LTO	2.33667	5.40748(2)	5.41297(2)	13.08390(3)	0.059(3)	0.0289(5)	0.937(3)	0.905(10)	1.44(2)
350	LTO	2.39773	5.40858(2)	5.41205(2)	13.08697(2)	0.061(3)	0.0277(5)	0.969(3)	0.982(10)	1.45(2)
360	LTO	2.40479	5.40950(1)	5.41173(2)	13.08996(2)	0.050(4)	0.0241(5)	1.012(3)	0.997(10)	1.34(2)
370	HTT	2.69404	5.41071(1)	5.41071(1)	13.09232(3)	0	0.2249(6)	1.036(3)	1.03(1)	1.46(2)
380	HTT	2.71791	5.41123(1)	5.41123(1)	13.09454(3)	0	0.2095(6)	1.047(3)	0.91(1)	1.49(2)
390	HTT	2.57769	5.41176(1)	5.41176(1)	13.09686(2)	0	0.2217(6)	1.078(3)	0.96(1)	1.38(2)
400	HTT	2.49765	5.41237(1)	5.41237(1)	13.09893(2)	0	0.2135(6)	1.110(3)	0.99(1)	1.41(2)

Table 4.5: Refined parameters from Rietveld refinements against variable temperature XRD patterns of $La_{1.875}Ba_{0.125}(Cu_{1-y}Mg_y)_{0.875}Cu_{0.125}O_4$ y = 0.4.

Temperature (K)	Phase	R_{wp}	a (Å)	b (Å)	c (Å)	X_3^+	Γ_1^+	$B_{iso}(A)$ (Å^2)	$B_{iso}(B)$ (Å^2)	$B_{iso}(O)$ (Å^2)
190	LTO	2.69526	5.40560(1)	5.43326(2)	13.00070(3)	0.104(2)	0.0259(6)	1.056(3)	0.99(1)	1.16(2)
200	LTO	2.72053	5.40591(1)	5.43319(2)	13.00303(3)	0.103(2)	0.0264(6)	1.076(4)	1.00(1)	1.15(2)
210	LTO	2.69908	5.40642(1)	5.43312(2)	13.00503(3)	0.099(2)	0.0263(6)	1.094(4)	1.02(1)	1.17(2)
220	LTO	2.71927	5.40695(1)	5.43285(2)	13.00736(3)	0.097(2)	0.0261(6)	1.112(4)	1.04(1)	1.15(2)
230	LTO	2.72489	5.40748(1)	5.43267(2)	13.00919(3)	0.096(2)	0.0262(6)	1.127(4)	1.04(1)	1.15(2)
240	LTO	2.71178	5.40822(1)	5.43233(2)	13.01172(3)	0.095(2)	0.0269(6)	1.146(4)	1.05(1)	1.14(2)
250	LTO	2.70724	5.40891(1)	5.43199(2)	13.01409(3)	0.094(2)	0.0275(6)	1.157(4)	1.06(1)	1.16(2)
260	LTO	2.69777	5.40965(1)	5.43165(2)	13.01633(3)	0.092(2)	0.0279(6)	1.164(4)	1.07(1)	1.18(2)
270	LTO	2.69502	5.41042(1)	5.43131(2)	13.01862(3)	0.091(2)	0.0277(6)	1.175(4)	1.08(1)	1.22(2)
280	LTO	2.71093	5.41116(1)	5.43082(2)	13.02124(3)	0.089(2)	0.0273(6)	1.191(4)	1.08(1)	1.21(2)
290	LTO	2.70317	5.41198(1)	5.43042(2)	13.02359(3)	0.087(2)	0.0268(6)	1.210(4)	1.09(1)	1.27(2)
300	LTO	2.72726	5.41270(1)	5.43003(2)	13.02571(3)	0.086(2)	0.0275(6)	1.220(4)	1.11(1)	1.28(2)
310	LTO	2.75832	5.41363(1)	5.42942(2)	13.02857(3)	0.083(2)	0.0288(6)	1.240(4)	1.13(1)	1.29(2)
320	LTO	2.7393	5.41543(1)	5.42828(2)	13.03394(3)	0.077(3)	0.0313(6)	1.271(4)	1.15(1)	1.36(2)

330	LTO	2.71494	5.41661(2)	5.42748(2)	13.03707(3)	0.075(3)	0.0325(6)	1.290(4)	1.15(1)	1.31(2)
340	LTO	2.7409	5.41784(2)	5.42660(2)	13.04010(3)	0.074(3)	0.0299(6)	1.313(4)	1.22(1)	1.36(2)
350	LTO	2.7852	5.41921(2)	5.42548(3)	13.04356(3)	0.069(3)	0.0310(6)	1.313(4)	1.28(1)	1.28(2)
360	LTO	2.77819	5.42058(2)	5.42454(3)	13.04673(3)	0.068(3)	0.0281(6)	1.327(4)	1.29(1)	1.34(2)
370	LTO	2.84937	5.42148(2)	5.42414(3)	13.04946(3)	0.069(3)	0.0261(6)	1.356(4)	1.30(1)	1.33(2)
380	LTO	2.91085	5.42223(2)	5.42422(3)	13.05190(3)	0.066(3)	0.0220(6)	1.387(4)	1.31(1)	1.36(2)
390	HTT	3.07255	5.42328(1)	5.42328(1)	13.05433(3)	0	0.2390(7)	1.397(4)	1.31(1)	1.53(2)
400	HTT	3.06088	5.42387(1)	5.42387(1)	13.05433(3)	0	0.2417(7)	1.432(4)	1.28(1)	1.57(2)

Table 4.6: Refined parameters from Rietveld refinements against variable temperature XRD patterns of $La_{1.875}Ba_{0.125}(Cu_{1-y}Mg_y)_{0.875}Cu_{0.125}O_4$ y = 0.5.

5. Predicting T_{LTO} from tolerance factor, doping level, and A-site variance

Temperatures for the LTO-to-HTT phase transition were gathered from a series of publications and are reported here with their references and method of experiment:

$La_{2-x}Sr_xCuO_4$			
x	T_{LTO} (K)	*Reference*	*Technique*
0.001	527.530	Kato et al. 1988	Powder X-ray diffraction
0.021	506.883	Kato et al. 1988	Powder X-ray diffraction
0.041	459.514	Kato et al. 1988	Powder X-ray diffraction
0.061	449.798	Kato et al. 1988	Powder X-ray diffraction
0.100	285.830	Kato et al. 1988	Powder X-ray diffraction
0.113	289.474	Takagi et al. 1992	Powder X-ray diffraction
0.150	186.235	Takagi et al. 1992	Powder X-ray diffraction
0.151	178.947	Radaelli et al. 1994	Neutron powder diffraction
0.170	141.296	Radaelli et al. 1994	Neutron powder diffraction
0.179	115.789	Radaelli et al. 1994	Neutron powder diffraction
0.189	97.571	Radaelli et al. 1994	Neutron powder diffraction
0.201	63.563	Radaelli et al. 1994	Neutron powder diffraction
0.205	39.271	Radaelli et al. 1994	Neutron powder diffraction
0.207	21.053	Radaelli et al. 1994	Neutron powder diffraction
0.209	21.053	Takagi et al. 1992	Powder X-ray diffraction

$La_{2-x}Ba_xCuO_4$			
x	T_{LTO} (K)	*Reference*	*Technique*
0.100	313.150	Hücker 2011	Single crystal XRD
0.116	274.851	Hücker 2011	Single crystal XRD
0.126	250.914	Hücker 2011	Single crystal XRD
0.130	241.339	Hücker 2011	Single crystal XRD
0.136	226.977	Hücker 2011	Single crystal XRD
0.150	193.465	Hücker 2011	Single crystal XRD

$La_{1.875}Ba_{0.125}(Cu_{1-y}Mg_y)_{0.875}Cu_{0.125}O_4$			
x	T_{LTO} (K)	*Reference*	*Technique*
0	254	This work	High resolution XRD
0.1	276	This work	High resolution XRD
0.2	291	This work	High resolution XRD
0.3	332	This work	High resolution XRD
0.4	366	This work	High resolution XRD
0.5	380	This work	High resolution XRD

$La_{2-x}Nd_{0.4}Sr_xCuO_4$			
x	T_{LTO} (K)	*Reference*	*Technique*
0.169	291.148	Dragomir et al. 2020.	Single crystal X-ray diffraction
0.190	272.763	Dragomir et al. 2020.	Single crystal X-ray diffraction
0.200	250.335	Axe and Crawford, 1994.	Synchrotron X-ray powder diffraction
0.240	149.007	Dragomir et al. 2020.	Single crystal X-ray diffraction
0.250	129.058	Axe and Crawford, 1994.	Synchrotron X-ray powder diffraction

$\langle r_A \rangle = 1.223$ Å				
$L_{1.85}M_{0.15}CuO_4$	A-site variance	T_{LTO} (K)	*Reference*	*Technique*
$La_{0.925}Sr_{0.075}$	0.000539	192.677	McAllister and Attfield, 2002.	Powder neutron diffraction
$La_{0.925}Sr_{0.060}Ca_{0.008}Ba_{0.007}$	0.000816	196.212	McAllister and Attfield, 2002.	Powder neutron diffraction
$La_{0.925}Sr_{0.045}Ca_{0.017}Ba_{0.013}$	0.001083	220.960	McAllister and Attfield, 2002.	Powder neutron diffraction
$La_{0.925}Sr_{0.030}Ca_{0.025}Ba_{0.020}$	0.001359	234.217	McAllister and Attfield, 2002.	Powder neutron diffraction
$La_{0.925}Sr_{0.008}Ca_{0.037}Ba_{0.030}$	0.001806	253.662	McAllister and Attfield, 2002.	Powder neutron diffraction
$La_{0.900}Nd_{0.025}Ca_{0.037}Ba_{0.038}$	0.002263	281.503	McAllister and Attfield, 2002.	Powder neutron diffraction
$La_{0.875}Nd_{0.050}Ca_{0.032}Ba_{0.043}$	0.002627	301.831	McAllister and Attfield, 2002.	Powder neutron diffraction

$\langle r_A \rangle = 1.232$ Å				
$L_{1.85}M_{0.15}CuO_4$	A-site variance	T_{LTO} (K)	*Reference*	*Technique*
$La_{0.925}Sr_{0.019}Ba_{0.056}$	0.003503	210.354	McAllister and Attfield, 2002.	Powder neutron diffraction
$La_{0.921}Nd_{0.004}Sr_{0.018}Ba_{0.057}$	0.003594	213.889	McAllister and Attfield, 2002.	Powder neutron diffraction
$La_{0.906}Nd_{0.019}Sr_{0.013}Ba_{0.062}$	0.003898	220.960	McAllister and Attfield, 2002.	Powder neutron diffraction
$La_{0.888}Nd_{0.037}Sr_{0.007}Ba_{0.068}$	0.004305	254.545	McAllister and Attfield, 2002.	Powder neutron diffraction
$La_{0.868}Nd_{0.058}Ba_{0.075}$	0.004701	271.338	McAllister and Attfield, 2002.	Powder neutron diffraction

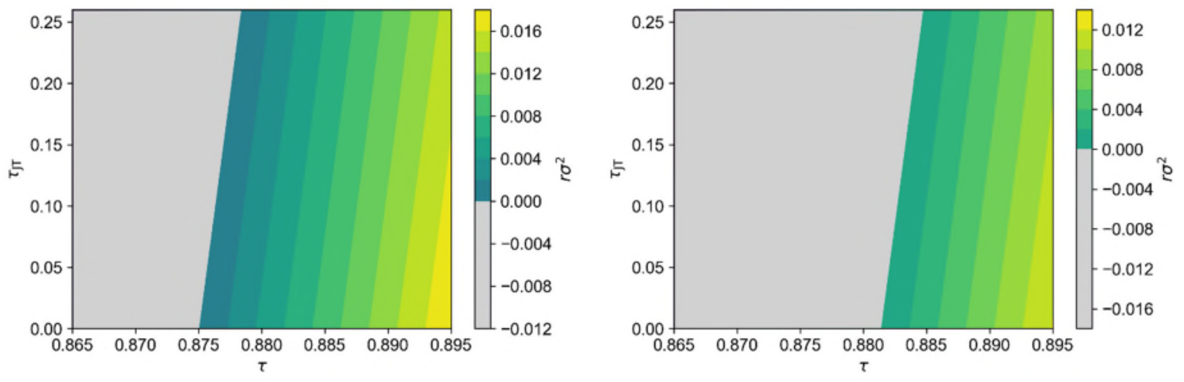

Figure 5.1: Isotherms T_{LTO} = 300 K (left) and 100 K (right) computed as a function tolerance factor (τ), doping level (τ_{JT}), and A-site variance ($r\sigma^2$). Physically inaccessible regions on account of negative A-site variances are shaded in grey.

Justification for updated Ni^{2+} atomic radii value:

First we consider first the crystallographic structure of La_2MgO_4 at 990 K, well above the LTO-to-HTT transition temperature. The average Mg-O bond length can be calculated from the cif file reported by Tidey et al. 2022, as 2.0660 Å. The Shannon ionic radii (Shannon, 1976) for two-coordinate O^{2-} is 1.35 Å. We then calculate the Mg ionic radii as 2.0660 Å – 1.35 Å = 0.716 Å. The reported Shannon value for the six-coordinate ionic radii is 0.72 Å.

We then perform the same calculation for the La_2NiO_4 system at 1073 K (i.e. similarly high temperature to the above) . The average bond length calculated from Skinner 2003 is 2.0620 Å. Subtracting the radii for O^{2-}: 2.0620 Å – 1.35 Å = 0.712 Å.

We correct this value by an offset (reflecting that the values tabulated by Shannon for ambient conditions will differ from those we have calculated at ~ 1000K) calculated from the previous Mg–O calculation:

(0.72/0.716)*0.712 = **0.716 Å**

It is important to note this is not a true prediction since the ionic radii is determined from experimental data, albeit from a single crystal structure well above the phase transition temperature.

References

Axe, J. D., & Crawford, M. K. (1994). Structural Instabilities in Lanthanum Cuprate. *Journal of Low Temperature Physics*, *95*, 271–284.

Campbell, B. J., Stokes, H. T., Tanner, D. E., & Hatch, D. M. (2006). ISODISPLACE : a web-based tool for exploring structural distortions. *Journal of Applied Crystallography*, *39*, 607–614.

Coelho, A. A. (2018). TOPAS and TOPAS-Academic: An optimization program integrating computer algebra and crystallographic objects written in C++: An. *Journal of Applied Crystallography*, *51*(1), 210–218.

Dove, M. T. (1997). Theory of displacive phase transitions in minerals. *American Mineralogist*, *82*(3–4), 213–244.

Dragomir, M., Ma, Q., Clancy, J. P., Ataei, A., Dube, P. A., Sharma, S., Huq, A., Dabkowska, H. A., Taillefer, L., & Gaulin, B. D. (2020). Materials preparation, single-crystal growth, and the phase diagram of the cuprate high-temperature superconductor $La_{1.6-x}Nd_{0.4}Sr_xCuO_4$. *Physical Review Materials*, *4*(11).

Hord, R., Cordier, G., Hofmann, K., Buckow, A., Pascua, G., Luetkens, H., Alff, L., & Albert, B. (2011). Transitions between lanthanum cuprates: Crystal structures of T', orthorhombic, and K_2NiF_4-type La_2CuO_4. *Zeitschrift Fur Anorganische Und Allgemeine Chemie*, *637*(9), 1114–1117.

Hücker, M., v. Zimmermann, M., Gu, G. D., Xu, Z. J., Wen, J. S., Xu, G., Kang, H. J., Zheludev, A., & Tranquada, J. M. (2011). Stripe order in superconducting $La_{2-x}Ba_xCuO_4$ ($0.095 \leq x \leq 0.155$). *Physical Review B*, *83*(10), 41–50.

Kato, M., Maeno, Y., & Fujita, T. (1988). Variation of temperature-linear specific heat with doping in $(La_{1-x}Sr_x)_2CuO_4$. *Physica C: Superconductivity and Its Applications*, *152*(1), 116–120.

McAllister, J. A., & Attfield, J. P. (2002). Cation size control of structure, structural fluctuations, and superconductivity in (formula presented). *Physical Review B - Condensed Matter and Materials Physics*, *66*(1), 1–10.

Radaelli, P. G., Hinks, D. G., Mitchell, A. W., Hunter, B. A., Wagner, J. L., Dabrowski, B., Vandervoort, K. G., Viswanathan, H. K., & Jorgensen, J. D. (1994). Structural and superconducting properties of $La_{2-x}Sr_xCuO_4$ as a function of Sr content. *Physical Review B*, *49*(6), 4163–4175.

Shannon, R. D. (1976). Revised Effective Ionic Radii and Systematic Studies of Interatomic Distances in Halides and Chalcogenides. *Foundations of Crystallography,* 32(5)*, 751-767.*

Skinner, S.J.; (2003) Characterisation of La2 Ni O4+d using in-situ high temperature neutron powder diffraction. Solid State Sciences, 5, 419-426.

Stephens, P. W. (1999). Phenomenological model of anisotropic peak broadening in powder diffraction. *Journal of Applied Crystallography*, *32*(2), 281–289.

Takagi, H., Cava, R. J., Marezio, M., Batlogg, B., Krajewski, J. J., Peck, W. F., Bordet, P., & Cox, D. E. (1992). Disappearance of superconductivity in overdoped $La_{2-x}Sr_xCuO_4$ at a structural phase boundary. *Physical Review Letters*, *68*(25), 3777–3780.

Tidey, J. P., Keegan, C., Bristowe, N. C., Mostofi, A. A., Hong, Z. M., Chen, B. H., Chuang, Y. C., Chen, W. T., & Senn, M. S. (2022). Structural origins of the low-temperature orthorhombic to low-temperature tetragonal phase transition in high-T_c cuprates. *Physical Review B*, *106*(8), 085112(6).

van Vleck, J. H. (1939). The Jahn-Teller Effect and Crystalline Stark Splitting for Clusters of the Form XY6. *The Journal of Chemical Physics*, *7*, 72–84.
